# Association of TRB3 Q84R polymorphism with polycystic ovary syndrome in Chinese women

**DOI:** 10.1186/1477-7827-9-46

**Published:** 2011-04-14

**Authors:** Xue Zhang, Li Fu, Qiufang Zhang, Liying Yan, Yanmin Ma, Binbin Tu, Nana Liu, Jie Qiao

**Affiliations:** 1 Department of Obstetrics and Gynecology, Peking University Third Hospital, Beijing 100191, P.R China; 2 Reproductive Medical Center, Beijing Obstetrics and Gynecology Hospital, Capital Medical University, Beijing 100026, P.R China

## Abstract

**Background:**

Tribbles 3 (TRB3) affects insulin signalling by inhibiting insulin-stimulated Akt phosphorylation and subsequent activation. A single nucleotide polymorphism located in the second extron of the human TRB3 gene is thought to be associated with insulin resistance. The latter is a core abnormality in PCOS independent of obesity. The present study was designed to clarify the relationships of TRB3 Q84R polymorphism with PCOS in a Chinese women group.

**Methods:**

A case-control study with two groups: PCOS group (n = 336) and control group of infertility women for tubal and/or male factor (n = 116) was performed. Genotyping of the TRB3 R84 variant was determined by polymerase chain reaction-restriction fragment length polymorphism (PCR-RFLP).

**Results:**

The frequency of genotype QQ in PCOS women was significantly lower, while genotype QR and RR were significantly higher than that in control group (p < 0.05). However, the difference disappeared after adjustment for BMI. At glucose1h, glucose2h and insulin2h point, the difference between QQ individuals and R84 allele carriers in PCOS women reached statistical significance during OGTT (p < 0.05).

**Conclusions:**

TRB3 Q84R polymorphism is associated with obesity and especially glucose metabolism and not associated with polycystic ovary syndrome because of compositional characteristics of phenotype in Chinese PCOS women.

## Background

Polycystic ovary syndrome (PCOS) is a common endocrine disorder with amenorrhoea (or oligomenorrhoea), hyperandrogenism, hirsutism, obesity and insulin resistance (IR). It affects 5-10% reproductive aged women and is a leading cause of female infertility [1]. It is a complex and heterogeneous disorder due to the interaction of multiple genetic defects and environmental factors [2]. Candidate genes for etiology of PCOS involve in ovarian and adrenal steroidogenesis, steroid hormone actions, gonadotropin action and regulation, insulin action and secretion, energy homeostasis, chronic inflammation and others [3]. Insulin resistance is a pathophysiological contributor in around 50% to 80% of women with PCOS [4]. It is well established that hyperinsulinemia and insulin resistance are common biochemical features of PCOS independent of obesity [5]. Insulin resistance is associated with hyperandrogenemia and anovulation in PCOS women [6,7]. As is well known, hyperandrogenism and anovulation are important clinical features and diagnostic criteria for polycystic ovary syndrome [8]. Besides, PCOS women are at substantial risks for development of glucose intolerance, diabetes, lipid abnormalities and cardiovascular abnormalities since they often have insulin resistance [6].

Tribbles 3 (TRB3), a mammalian homolog of Drosophila tribbles also known as TRIB3/NIPK (gene ID 57761), affects insulin signalling by inhibiting insulin-stimulated Akt phosphorylation. Literature has shown that mice with overexpressing TRB3 had increased liver glucose output and become hyperglycemic [9]. The expression of TRB3 mRNA and protein increased in skeletal muscle and liver from female rat offspring which were insulin-resistant diabetes [10,11]. In human, hepatic mRNA expression of TRB3 was significantly increased in obese people with insulin resistance [12]. TRB3 protein level in skeletal muscle significantly elevated in type 2 diabetes mellitus (T2DM) patients [13]. Studies had also shown that TRB3 Q84R polymorphism was associated with insulin resistance and related cardiovascular risk [14-18]. In terms of mechanism for the above phenomenon, mice overexpressing TRB3 R84 in beta cells displayed decreased beta cell mass [19]. All the above results suggested that TRB3 was associated with insulin resistance. Yet this hypothesis has not been tested in PCOS patients.

The current study aims at examining the relationships of TRB3 Q84R polymorphism with insulin resistance in a group of Chinese women patients with PCOS.

## Methods

### Subjects

The PCOS patients in this study were consisted of 336 infertile women aged 17-40. The diagnosis of PCOS was based on the Rotterdam criteria [8]. Two out of three of the following criteria were required for the diagnosis: oligo-ovulation and/or anovulation, clinical and/or biochemical signs of hyperandrogenism and polycystic ovaries (by transvaginal ultrasound). Patients with the following disorders were excluded: hyperprolactinemia, nonclassic congenital adrenal hyperplasia, Cushing's syndrome, androgen-secreting neoplasms and thyroid dysfunction. Oligo-ovulation and/or anovulation were defined by the presence of oligomenorrhea or amenorrhea respectively. Hyperandrogenism was defined as the clinical presence of hirsutism (Ferriman-Gallwey score ≥6), acne, or alopecia and/or elevated androgen levels according to normal reference values (total serum testosterone >2.8 nmo/l). Polycystic ovarian morphology was examined by trans-vaginal ultrasound and polycystic ovary was defined by presence of 12 or more follicles in each ovary measuring 2-9 mm in diameter, and/or increased ovarian volume (>10 ml) during the follicular phase of a menstrual cycle. A diagnosis of PCOS was made only after prolactin, FSH, E_2_, TSH, and 17-hydroxyprogesterone (17-HP) levels had been measured to exclude hyperprolactinemia, premature ovarian failure, hypothalamic amenorrhea, thyroid disorders, and 21-hydroxylase-deficient nonclassic adrenal hyperplasia (NCAH), respectively. Cushing's syndrome and androgen-secreting neoplasms were excluded by clinical presence, history and laboratory examinations.

The control group included 116 age-matched women with regular menstrual cycles who were infertile for tubal and/or male factor. They have no polycystic ovaries and no clinical or biochemical signs of hyperandrogenism.

None of the patients had taken any medications affecting glucose metabolism during the preceding 3 months. The study was approved by the institutional ethics committee, and written informed consent was obtained from each patient.

### Hormonal and biochemical measures

Fasting blood samples were taken during the follicular phase of a menstrual cycle (spontaneous or bleeding after progestin withdrawal) of all women. Luteinizing hormone (LH), follicle stimulating hormone (FSH) and total serum testosterone concentrations (T) were determined by chemiluminescence immunoassay. Total cholesterol (CHO) and triglycerides (TG) were determined by oxidase method, HDL-cholesterol (HDL) by synthetic polymer/detergent HDL-C assay (SPD method) and LDL-cholesterol (LDL) by surfactant LDL-C assay (SUR method).

A 75 g oral glucose tolerance test was performed in each PCOS woman. Blood was obtained for glucose and insulin determinations at 0 h, 1 h and 2 h after glucose load. Glucose levels were detected using the glucose oxidase method and insulin levels were determined by chemiluminescence immunoassay. Insulin resistance (IR) was assessed by the homeostasis model assessment (HOMA), which was calculated as [fasting insulin concentration (μIu/ml) × fasting glucose concentration(mmol/l]/22.5 [20]. Body mass index (BMI) was calculated as body weight (kg) divided by body height squared (m^2^).

### Genotyping

Genomic DNA was extracted from peripheral blood leukocytes using the standard salting-out method [21]. The purity and concentration of the isolated DNA was measured. Genotyping of the TRB3 R84 variant was performed by polymerase chain reaction-restriction fragment length polymorphism (PCR-RFLP) as previously described [14]. Five percent of samples from both PCOS and control women were re-genotyped by other laboratory personnel and no discrepancy in genotyping was noticed.

### Statistical analysis

The Kolmogorov-Smirnov test was used to test for normal distribution. Comparisons of continuous variables between groups were conducted by unpaired student's t test. Mann-Whitney U-test was used when continuous variables did not meet the normal distribution. The Chi-square test was used to analyze the associations between categorical variables. All analyses were performed by SPSS software version 17.0 (SPSS Inc. Chicago, IL, USA). Tests of statistical significance were two-sided and taken as significant when P < 0.05.

## Results

The basal demographical, hormonal and biochemical parameters of control and PCOS women are summarized in Table 1. As expected, significantly higher levels of BMI, T, LH/FSH ratio, fasting glucose, CHO, TG and LDL were found in PCOS group compared with control group while the levels of HDL were significantly lower in PCOS women (p < 0.05).

**Table 1:** Clinical and endocrine-metabolic parameters in control and PCOS women

	control (n = 116)	PCOS (n = 336)
Age(yr)	30 (21-41)	29 (18-40)
BMI(kg/m^2^)	22 (16-29)	24 (16-35)*
LH/FSH	0.5 (0.2-2.1)	1.3 (0.1-6.4)*
T(nmol/l)	0.7 (0.02-1.9)	1.7 (0.02-7)*
Glu0(mmol/l)	4.8 (3.7-6.0)	5.0 (3.5-8.7)*
CHO(mmol/l)	4.3 (2.7-7.2)	4.9 (3.0-7.8)*
TG(mmol/l)	0.9 (0.3-3.2)	1.3 (0.4-5.9)*
HDL(mmol/l)	1.3 (0.8-2.2)	1.2 (0.7-2.3)*
LDL(mmol/l)	2.4 (1.0-5.0)	2.8 (0.6-6.2)*

Mann-Whitney U-test; Data are described in median (range); BMI:body mass index; LH:luteinizing hormone; FSH: follicle stimulating hormone; T:total serum testosterone;Glu0:fasting glucose; CHO:total cholesterol; TG:triglycerides; HDL: HDL-cholesterol; LDL: LDL-cholesterol. *: p < 0.05.

### Comparison of TRB3 Q84R polymorphism between PCOS and control women

**Figure 1:**
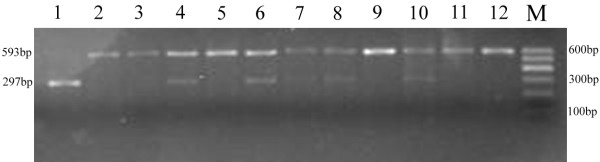
**The representative electrophoretic gel featuring the RFLP analysis of TRB3 Q84R polymorphism**. The PCR products were digested with MspІ followed by 2% agarose gel electrophoresis: the resulting wild-type (QQ) samples contained one band sized 593 bp, heterozygotes (QR) samples contained two bands sized 593 bp and 297 bp and homozygotes (RR) contained one band sized 297 bp. M: 100 bp DNA marker; 1: RR genotype; 2,3,5,9,11,12: QQ genotype; 4,6,7,8,10: QR genotype.

Genotyping for the TRB3 Q84R polymorphism was shown in Figure 1. The genotype frequencies were in agreement with the Hardy-Weinberg equilibrium (P > 0.05 for all analyses). Genotype frequency was significantly different between PCOS and control women (Table 2). The frequencies of R allele were 19.5% in PCOS women and 12.5% in control, and Q allele were 80.5% and 87.5% respectively (P = 0.016). However, the significant difference disappeared after adjustment for BMI by dividing patients into four subgroups according to BMI (Asia-Pacific criteria: underweight: <18.5 kg/m^2^; normal weight: 18.5-22.9 kg/m^2^; overweight: 23.0-24.9 kg/m^2^; obese: ≥25.0 kg/m^2^).

**Table 2:** TRB3 Q84R genotype distribution in PCOS and control women

genotype	PCOS	control	P
	n = 336	n = 116	
QQ	209 (62.2%)	88 (75.9%)	0.02
QR	123 (36.6%)	27 (23.3%)	
RR	4 (1.2%)	1 (0.9%)	
alleles			
Q	541 (80.5%)	203 (87.5%)	0.016
R	131 (19.5%)	29 (12.5%)	

Chi-square test; Data are presented as number and (percentage).

### Comparison of TRB3 Q84R polymorphism between PCOS women with IR and without IR

PCOS group was divided into insulin resistance group (PR group) and non-insulin resistance group (PNR group) according to HOMA-IR, with a threshold value of 2.69 [22]. In PCOS women, the frequencies of QR and RR genotypes in non-insulin resistance group were lower than insulin resistance group while QQ genotype was higher, but with no statistical significance ( see Table 3).

**Table 3:** TRB3 Q84R genotype distribution in PCOS women with IR and without IR

genotype	PR	PNR	P
	n = 143	n = 193	
QQ	81 (56.6%)	128 (66.3%)	0.104
QR	59 (41.3%)	64 (33.2%)	
RR	3 (2.1%)	1 (0.5%)	

Chi-square test; Data are presented as number and (percentage). IR: insulin resistance. PR: PCOS with insulin resistance; PNR: PCOS without insulin resistance.

### TRB3 Q84R polymorphism and main clinical features of PCOS women

In further analysis, PCOS women were divided into hyperandrogenism group and non-hyperandrogenism group according to serum total testosterone concentration. TRB3 Q84R genotype distribution is shown in Table 4. The frequencies of QR and RR genotypes in non-hyperandrogenism group were lower while QQ genotype was higher than hyperandrogenism group, though not significant. Similar tendency of genotype distribution was observed between oligo/anovulation group and ovulation group in PCOS women, which are shown in Table 5.

**Table 4:** TRB3 Q84R genotype distribution in PCOS women divided into hyperandrogenism group and non-hyperandrogenism group

genotype	HA	NHA	P
	n = 65	n = 271	
QQ	34 (52.3%)	175 (64.6%)	0.12
QR	30 (46.2%)	93 (34.3%)	
RR	1 (1.5%)	3 (1.1%)	

Chi-square test; Data are presented as number and (percentage). HA: hyperandrogenism; NHA: non-hyperandrogenism

**Table 5:** TRB3 Q84R genotype distribution in PCOS women divided into oligo/anovulation group and ovulation group

genotype	oligo/anovulation	ovulation	P
	n = 307	n = 29	
QQ	189 (61.6%)	20 (69%)	0.687
QR	114 (37.1%)	9 (31%)	
RR	4 (1.3%)	0 (0%)	

Chi-square test; Data are presented as number and (percentage). oligo/anovulation: cycle intervals > 35 days or absence of menstruation for 3 consecutive months; ovulation: cycle intervals ≤ 35 days

### TRB3 Q84R polymorphism and glucose metabolism of PCOS women

Because of the small number of RR individuals, subjects with QR and RR genotypes were considered together as R84 carriers. The endocrine and metabolic characteristics of PCOS women by TRB3 Q84R genotype are shown in Table 6. At each time point of OGTT, the median values of glucose levels were higher in R84 carriers than QQ individuals. The similar trend was also observed for median values of insulin levels. At glucose1h, glucose2h and insulin2h point, the difference between genotype QQ individuals and R84 carriers reached statistical significance. Regarding to TRB3 Q84R polymorphism in PCOS women, there was a stepwise increase in BMI and HOMA-IR from Q84Q to Q84R and R84R genotype, although not significant.

**Table 6:** Clinical and endocrine-metabolic characteristics of PCOS women according to TRB3 Q84R genotype

	QQ (n = 209)	QR+ RR (n = 127)
Age(yr)	28 (17-40)	28 (19-38)
BMI(kg/m^2^)	24 (16-33)	25 (17-35)
LH/FSH	1.27 (0.01-5.2)	1.48 (0.22-6.4)
T(nmol/l)	1.6 (0.21-7.11)	1.7 (0.02-6.14)
HOMA-IR	2.2 (0.4-12.3)	2.6 (0.4-12.8)
glu0h(mmol/l)	4.8 (3.5-8.7)	4.9 (3.7-8.0)
glu1h(mmol/l)	7.4 (3.3-17.9)	8.2 (3.4-60.1)*
glu2h(mmol/l)	6.2 (3.5-16.8)	6.6 (3.4-50.8)*
Ins0h(μIU/ml)	9.7 (2.0-56.6)	11.8 (2.0-45.8)
Ins1h(μIU/ml)	74.6 (1.2-300)	86.8 (7.1-300)
Ins2h(μIU/ml)	67.3 (6.8-300)	76.5 (6.7-300)*

Mann-Whitney U-test; Data are described in median (range); HOMA-IR: homeostatic model assessment of insulin resistance;glu:glucose; Ins:insulin. *: p < 0.05.

## Discussion

The spectrum of evidence for PCOS genetic basis is very board. Molecular defects in gonadotrophins and their receptors, in enzymes involved in steroidogenesis, as well as those contributed to insulin resistance and its sequelae, have been the principal focus of family-based linkage analysis and case-control studies. The common occurrence of insulin resistance and pancreatic β-cell dysfunction in association with PCOS and the increased risk for development of T2DM is now well recognized [23,24]. Moreover, insulin acting through its receptor stimulates steroidogenesis. This has led investigations to focus on insulin resistance in PCOS [6]. Gene variants related to insulin resistance such as insulin gene (VNTR), insulin receptor (INSR), insulin receptor substrate proteins (IRS1/2) and calpain-10 have shown to be associated with PCOS and related metabolic abnormality [25-28]. Particular interest is the implications of TRB3 as a candidate gene for insulin resistance [29]. Given the fact that insulin resistance is one of the prominent features in PCOS, it is reasonable to investigate TRB3 polymorphism in PCOS. To the best of our knowledge, this is the first such attempt to analyze TRB3 Q84R polymorphism in polycystic ovary syndrome.

In terms of the possible mechanism of TRB3 implication in insulin resistence and hence in PCOS, studies has found that TRB3 is located on 20p13-p12 of human chromosomal region that has been confirmed to be associated with human type 2 diabetes [30]. It is reported that TRB3 impairs insulin signalling through the inhibition of Akt phosphorylation and plays a role in insulin resistance [9]. It has also been demonstrated that the prevalent TRB3 missense Q84R polymorphism is significantly associated with several insulin resistance-related abnormalities in two independent cohorts of nondiabetic individuals. In fact, serum insulin levels were significantly different across the three genotype groups and higher in R84R individuals independent of BMI [14]. In addition, one study of 716 T2DM patients showed that the insulin resistance-related cardiovascular risk significantly increased in R84R individuals. In view of these findings, TRB3 Q84R polymorphism might also play a role as an insulin-resistance related factor in the pathogenesis of PCOS.

In our research, genotype frequency of Q84R was significantly different between PCOS and control women. The frequency of the R allele was significantly higher in PCOS women, while Q allele was significantly lower. Study has been demonstrated that R84 carriers had an increased risk of early-onset T2DM [17]. When either Q84 or R84 TRB3 full-length cDNAs were transfected in human HepG2 hepatoma cell lines, as compared with control HepG2 cells, insulin-induced Ser473-Akt phosphorylation was reduced by 22% in Q84- and, of note, by 45% in R84-transfected cells [14]. Study in human umbilical vein endothelial cell (HUVEC) naturally carrying different TRB3 genotypes (QQ,QR or RR) showed that cells carrying the TRB3 R84 variant (i.e., either QR- or RR-cells) had an impaired insulin signalling and reduced insulin-induced nitric oxide (NO) production as compared to the QQ-cells [15]. In our study, the analysis was adjusted for BMI as those studies reported that TRB3 was associated with obesity [12]. The significant difference of genotype frequency between PCOS and control women failed to be detected after adjustment for BMI. Different genotype frequency in control and PCOS group due to different BMI, and lost significance after BMI adjustment partly confirm association of Q84R with obesity, although the genotype groups of PCOS patients did not differ significantly in BMI. In further analysis, there was no significant difference in the genotype frequency between PCOS women with IR and without IR. Furthermore, we could not find an association between the R84 variant and anovulation or elevated total testosterone levels, which are main clinical characteristics of PCOS associated with insulin resistance in PCOS women. In addition, our study shows that R84 carriers of PCOS women have higher LH/FSH ratios. This is in line with the well known fact that women with polycystic ovary syndrome appear to have an increased luteinizing hormone pulse frequency [31]. It is possible that TRB3 R84 variant may be implicated in abnormal secretion of LH by certain underlying mechanism in PCOS women. However, the difference was not significant. Taken together, our results suggest that TRB3 Q84R polymorphism is associated with obesity while not with PCOS and insulin resistance of PCOS in Chinese women.

Meanwhile, subjects with R84 variant had higher glucose1h, glucose2h, insulin2h levels than individuals with QQ genotype in PCOS women (p < 0.05), which suggested that R84 variant increased the risk of glucose metabolic abnormality in PCOS women. This is consistent with observation from 645 non-diabetic subjects that the mean glucose levels were significantly higher in R84 carriers than QQ individuals and the mean insulin levels were higher in R84 carriers than QQ individuals with no significance at each time point of OGTT [17]. Indeed, R84 carriers were found to be insulin resistant compared with QQ individuals when studied by euglycemic, hyperinsulinemic clamp, a method that does not rely on endogenous insulin production for measuring insulin sensitivity [14]. Besides, the Q84R variant was associated with higher fasting insulin and higher HOMA-IR in T2DM group in Chinese population [16]. In the present study, there was a trend towards higher BMI and HOMA-IR in R84 allele carriers in comparison with QQ individuals in PCOS women, although with no statistical significance (Table 6). The observation was in accordance with the study about metabolic syndrome [18]. It is not surprising considering that both the polycystic ovary syndrome and the metabolic syndrome share insulin resistance as a central pathogenetic feature [32]. Thus, combined with other results on non-PCOS individuals, it may be deduced that the R84 variant is associated with glucose metabolic abnormality and is not associated with PCOS.

The above results of our study should be put into the context of the fact that the spectrum of clinical features of PCOS is very diverse and heterogeneous. The phenotype varies widely depending on life stage, genotype, ethnicity and environmental factors. Until recently no universally accepted clinical definition existed for PCOS. In past, only the classic phenotype of chronic anovulation and hyperandrogenism was included in the PCOS diagnosis [33]. The Rotterdam criteria are more extensive, including patients with distinct clinical appearances besides classic phenotype of PCOS. In fact, according to these guidelines, PCOS includes four different phenotypes: [1] O+H+P (oligo- and/or anovulation+hyperandrogenism+ polycystic ovaries); [2] O+H; [3] H+P; [4] O+P. It has been reported that different phenotypes of PCOS are distinct in clinical and endocrine characteristics, especially for nonhyperandrogenic PCOS phenotype that may develop PCOS by a different pathogenetic pathway [34-36]. Oligoanovulatory patients with PCO but without hyperandrogenism have mild endocrine and metabolic features of PCOS. The prevalence of metabolic syndrome and insulin resistance was lower in this phenotype with normal androgens compared with other phenotypes [35,36]. Similar to above reports, the prevalence of insulin resistance was lowest in nonhyperandrogenic PCOS phenotype (data not shown) in our study. In addition, relative prevalence of each phenotype differs for different racial and ethnic populations and different regions [34-36]. The relative prevalence of four phenotype in the present study was 16.6% (56/336), 5.1% (17/336), 8.0% (27/336) and 70.2% (236/336). The pattern of relative prevalence of four phenotypes of PCOS in present study, e.g., the prevalence of nonhyperandrogenic PCOS phenotype (O+P) was the highest and classic phenotype (O+H) was the lowest, was consistent with the results from a large-scale Chinese population [35]. Thus it is suspected that diverse compositional characteristics of phenotype in Chinese PCOS women may account for this observation that TRB3 Q84R is not associated with PCOS in the current study and this certainly merit further investigations with better categorizations of PCOS population.

## Conclusions

Taking together, the current study shows that TRB3 Q84R polymorphism is associated with obesity and especially glucose metabolism while is not associated with polycystic ovary syndrome and insulin resistance of PCOS in Chinese women. Further studies with a greater number of cases and sub-classification of cases according to different phenotypes of PCOS are necessary to confirm these findings.

## Competing interests

The authors declare that they have no competing interests.

## Authors' contributions

XZ participated in the design of the study, carried out the experiment, performed the statistical analysis and drafted the manuscript. LF, YM, BT and NL participated in collection of blood samples. LF, QZ and LY were involved in revision of manuscript drafts. JQ contributed to the design of the experiment and was responsible for finalising manuscript. All authors read and approved the final manuscript.
